# Joining the dots: the role of brokers in nutrition policy in Australia

**DOI:** 10.1186/s12889-017-4217-8

**Published:** 2017-04-08

**Authors:** Katherine Cullerton, Timothy Donnet, Amanda Lee, Danielle Gallegos

**Affiliations:** 1 School of Exercise and Nutrition Sciences, Queensland University of Technology, Victoria Park Rd, Kelvin Grove, QLD 4059 Australia; 2 School of Management, Queensland University of Technology, 2 George St, Brisbane, QLD 4000 Australia; 3 School of Public Health and Social Work, Queensland University of Technology, Victoria Park Rd, Kelvin Grove, QLD 4059 Australia

**Keywords:** Nutrition policy, Policy making, Advocacy, Food industry, Social network analysis, Influence

## Abstract

**Background:**

Poor diet is the leading preventable risk factor contributing to the burden of disease in Australia. A range of cost-effective, comprehensive population-focussed strategies are available to address these dietary-related diseases. However, despite evidence of their effectiveness, minimal federal resources are directed to this area. To better understand the limited public health nutrition policy action in Australia, we sought to identify the key policy brokers in the Australian nutrition policy network and consider their level of influence over nutrition policymaking.

**Methods:**

A social network analysis involving four rounds of data collection was undertaken using a modified reputational snowball method to identify the nutrition policy network of individuals in *direct* contact with each other. Centrality measures, in particular betweenness centrality, and a visualisation of the network were used to identify key policy brokers.

**Results:**

Three hundred and ninety (390) individual actors with 1917 direct ties were identified within the Australian nutrition policy network. The network revealed two key brokers; a Nutrition Academic and a General Health professional from a non-government organisation (NGO), with the latter being in the greatest strategic position for influencing policymakers.

**Conclusion:**

The results of this social network analysis illustrate there are two dominant brokers within the nutrition policy network in Australia. However their structural position in the network means their brokerage roles have different purposes and different levels of influence on policymaking. The results suggest that brokerage in isolation may not adequately represent influence in nutrition policy in Australia. Other factors, such as direct access to decision–makers and the saliency of the solution, must also be considered.

**Electronic supplementary material:**

The online version of this article (doi:10.1186/s12889-017-4217-8) contains supplementary material, which is available to authorized users.

## Background

Poor diet is the leading preventable risk factor contributing to the burden of disease in Australia [1, 2]. A range of cost-effective, comprehensive population-focussed strategies are available to address these dietary-related diseases [3–5]. However, despite evidence of the effectiveness, minimal federal resources are directed to this area in Australia [6]. This lack of action is occurring within a policy space that is characterised by a range of diverse interest groups or actors vying to influence public health nutrition policy, including: many different sectors of the food and beverage industry; health and agricultural organisations; national, state and territory government departments of health; agriculture; trade; and consumer affairs; academics and popular media figures. In different ways and for different motives, these interest groups seek to influence Australian food and nutrition policy and what Australians eat [7]. To better understand the limited public health nutrition policy action in Australia, we examined the power and influence of the actors involved in the policymaking process.

Power is a contested concept amongst political science scholars and is often conceptualized in a range of different ways, including ideational, structural and relational [8]. Historically the most common understanding of power has been relational where power is defined as the ability to achieve desired results; this can occur through the utilisation of resources and/or influence over actors [9]. Actors are powerful if they manage to influence outcomes in a way that brings them closer to their ideal endpoints [8]. However, this can be achieved in a number of different ways. A traditional view of power and influence in policymaking is that it comes from the possession of important resources, such as positional power, for example a Chief Executive Officer, or personal resources including education level or charisma [10]. The relative possession of these resources is thought to provide actors with a means of coercion or influence over others. However, some policy network scholars believe this traditional view of resource-based power is limiting, as they believe power is inherently a structural phenomenon within a network [10–12]. Accordingly, the authors view power within a policy network as being built upon relationships between actors and the distance between actors and their resources.

Policy networks are linkages between government bodies and other actors involved in public policymaking [13]. The networks are defined by geographic scope, a substantive issue and may involve hundreds of active actors from all levels of government, multiple interest groups, the media and research institutions [14]. These actors compete for their specific policy objectives to be translated into government policy. Some policy networks are considered “politically charged”, that is, they contain both allies and adversarial actors vying for pre-eminence by both enhancing their own position while also potentially subverting another’s outcomes [15]. This is the case for the nutrition policy network in Australia [16, 17].

Mapping out a policy network using social network analysis allows for the investigation of sometimes less obvious or hidden patterns in relationships that transcend hierarchical structure and improve our understanding of an actor’s relative power [18]. It has been used to examine a range of topics areas in health including community health coalitions [19], physician collaborations [20], relationships between tobacco control partners [21] and identifying the most powerful actors in public health policymaking [22]. A network consists of ‘ties’ which are patterns of association that link actors together; they can include informal linkages, based on communication and trust, as well as links based on the traditional institutionalized structures of co-ordination [23]. These connections can determine an actor’s ability to project power, control information flows and attempts to influence political outcomes or other actors [11]. The way an individual is embedded in a policy network can impose constraints on the individual as well as offer them opportunities [24]. Opportunities and potentially influence can be gained by those individuals who are well-connected to other informed individuals through their ability to access larger stores of useful political information [25]. Those on the periphery of networks, whose ties link them mainly to other marginal individuals, will encounter inadequate quantities and qualities of information [26]. They are in uninformed, hence uninfluential, locations.

Within social network analysis a range of different measures can be employed to explore power and influence. Centrality measures in general and *betweenness centrality* in particular are commonly used to identify influential individuals within a policy network [18]. Those with the highest levels of betweenness centrality act as brokers as they occupy a potentially privileged position in the networks structure and are often assumed to have a decisive impact on policy outcomes [27]. Centrality measures come in many forms including *degree*, *closeness* and *betweenness* (see Table 1). The concept of *closeness* in the Australian nutrition policy network has been explored by the authors in a previous paper [16]. This paper will explore power gained by being a broker as well as *degree centrality* in the Australian nutrition policy network.

**Table 1:** Measures of centrality [24]

*Degree*: the more ties (direct connections) an actor has, the more power they (may) have. Actors who have more ties have greater opportunities because they have more choices.
*In-degree*: the number of ties that lead into the actor directly from others, that is, the number of respondents who identified a particular actor as influential.
*Out-degree*: the number of ties that lead out of the actor directly to others, that is, the number of others identified by an individual actor as influential.
*Closeness*: If an actor is able to reach other actors at shorter path lengths, particularly decision-makers they will have greater influence. This position means power can be exerted by direct bargaining and exchange. Actors who are able to reach other actors at shorter path lengths have greater power and capacity to influence.
*Betweenness*: If an actor lies between other actors, that is, they act as a ‘broker’ that others must go through to reach a different group of people; they are in a position of power.

For detailed formulae for each of the above-used measures, please refer to Knoke and Yang (2008) [28] or Wasserman and Faust (1994) [29]

### Degree centrality (in-degree and out-degree)

Power and influence can come from various sources within a network. One measure is how highly nominated an individual is by others in the network *(in-degree*). If an individual receives many nominations or ties (an indication of a relationship or interactions) from others, they are said to be prominent or highly visible and this may indicate their importance [24]. Individuals who are able to nominate connections with a high number of people (*out-degree*) are able to communicate with many others, or make others aware of their views. However, network theorists note that simply having many connections is only one way to be influential [30]. A person with fewer connections might have more ‘important’ connections than someone with a large number of connections. One connection can be more important than another in different ways and in different contexts. Some connections are better because they link to well-connected people [16], whereas others are more important because they bridge across otherwise separated sections of the network [30].

### Betweenness centrality

*Betweenness centrality* is a measure that identifies individuals (brokers) who bridge different parts of the network. It specifically measures the number of times an actor is on the shortest path between two other actors [31]. *Betweenness centrality* is the most prominent centrality measure used to study power and dominance, because it indicates an actor’s strategic position as a broker between other actors in the network, thus enabling the spread of information [32]. Policy brokers can connect subsystems when groups differ in their beliefs and conflict about policy preferences exists [18].

Other actors in the network come to rely on brokers for indirect access to resources beyond their reach [33]. The broker is pivotal within this configuration and profits from others’ reliance on them. In turn, the group that emerges around the broker benefits overall because the broker extends the group’s opportunities and available resources [34]. Network analysis has demonstrated that brokers can have a significant impact on decision-making and are thus able to shape outcomes decisively at critical policy junctures [18], hence betweenness centrality is the primary measure reported in this article.

## Methods

A summary of the methods used is provided below; a more detailed description of the methodology is described elsewhere [16]. The aim was to identify those individuals who occupied structural positions of privilege in the nutrition policy network in Australia. Privileged structural positions in a network include those actors with high *centrality*, in particular *betweenness centrality*, and those with relatively low path distance to decision-makers (compared to other actors in the network). A previously used modified reputational snowball method [35] was undertaken to identify the nutrition policy network of individuals in *direct* contact with each other. This process began with asking a seed sample of nine leaders from diverse backgrounds in the nutrition policymaking process to ‘list the people you regard as influential in nutrition policy in Australia’(see Additional file 1). A definition of influence was provided which required that those nominated could do one or more of the following: demonstrate a capacity to shape ideas about policy; initiate policy proposals; substantially change or veto other’s proposals; or substantially affect implementation of policy related to food and nutrition [35]. Survey participants were required to note whether they were in direct contact with those they nominated and how often this direct contact occurred. The lead author then contacted all the nominees and asked them the same question. This process occurred for four successive rounds as data saturation was reached at this point.

All names received and their relationships with others were entered into social network analysis software, NodeXL [36], for both network visualisation and calculating centrality measures. Centrality measures were explored using in-degree (number of nominations an individual receives from others), out-degree (number of nominations of others an individual provides) and betweenness centrality (measures the extent an individual lies on paths between other individuals). A visualisation of the network was undertaken using the Harel-Koren Fast Multiscale algorithm with a high level of repulsion between vertices (repulsion =20) to visualise the network and emphasise brokerage roles.

## Results

Two hundred and eighty three individuals were invited to participate in the study and 140 responded, providing a response rate of 49%. The response rates for the different professional sectors are provided in Table 2.

**Table 2:** Number of respondents in each profession and response rates

Profession	Number (Response rate %)
Bureaucrat	38 (55)
Academic	38 (57)
Non-Government Organisation	37 (54)
Food Industry	16 (64)
Political	6 (18)
Public figures (celebrities)	2 (25)
Journalists	3 (23)

**Fig. 1:**
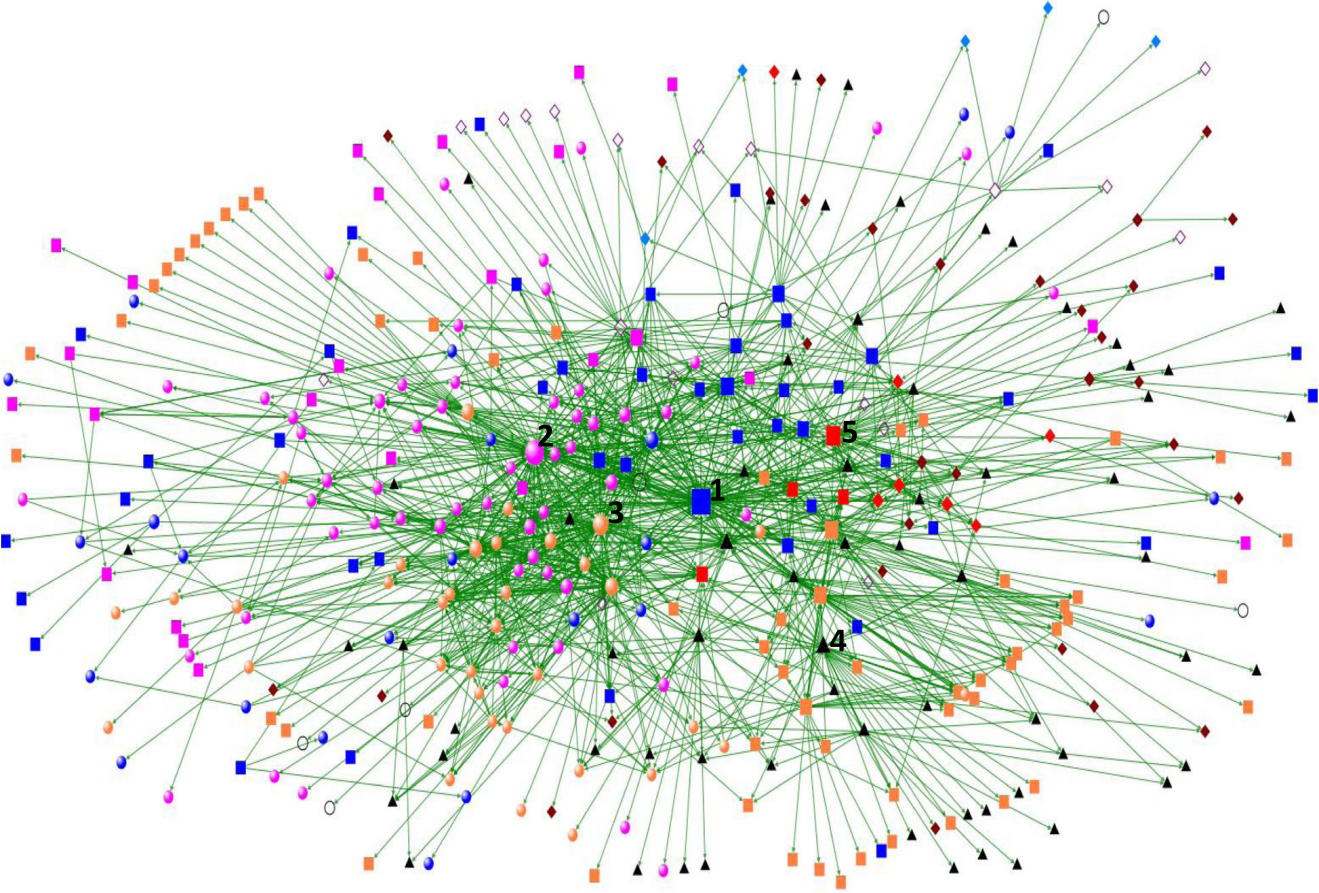
Network Analysis of the overall nutrition policy network in Australia. Key:  \  \  \  \ \ \. ◇ Public Figure\ ○ Nutrition Specialist \ □ General Health\ △ Private Sector

**Fig. 2:**
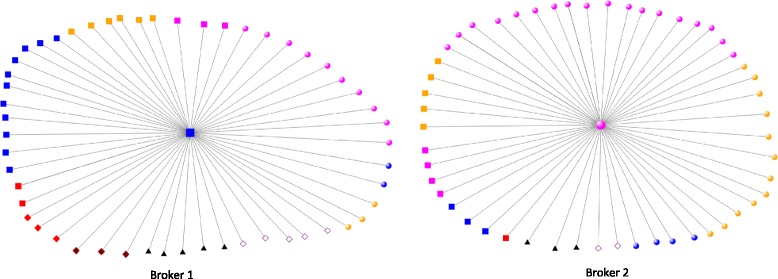
Direct ties from Brokers 1 and 2.Key:  \  \  \  \ \ \. ◇ Public Figure\ ○ Nutrition Specialist \ □ General Health\ △ Private Sector

Three hundred and ninety (390) individual actors with 1917 direct ties were identified in the nutrition policy network, with the network density described as relatively low at a measure of 0.009539 (potential actor relationships: actual actor relationships) and an average geodesic path distance of 3.29007 (7 maximum). Table 3 highlights the top five Brokers ranked by *betweenness centrality*, which as discussed above in Methods, is the lead indicator for power and dominance within the nutrition network. Brokers’ *degree*, *in-degree* and *out-degree* are also reported in Table 3 to provide additional context. Figure 1 presents a visual depiction of the overall nutrition policy network in Australia, which at a glance shows academic and government nutrition professionals congregating closely to Broker 2. Further analysis of the direct ties from Broker 2 confirm a higher proportion of direct ties with nutrition professionals compared to any other professional group (see Fig. 2). This aggregation of nutrition professionals around Broker 2 was also confirmed as a structural cluster within the network, via a cluster analysis conducted in a previous interrogation of the data set (see [16]).

**Table 3:** Betweenness centrality/degree centrality of nutrition policy network

Top 5 brokers ranked by betweenness centrality	Network location	Degree	Betweenness centrality	Normalised betweenness centrality	In-degree	Out-degree
General Health NGO	1	76	25,512	0.3381	49	53
Nutrition Professional Academic	2	77	24,039	0.3185	45	52
Nutrition Professional Government	3	60	13,930	0.1846	19	52
Food Industry	4	40	12,017	0.1592	3	40
General Health Government (decision-maker)	5	30	10,768	0.1427	10	24

In the present analysis, we find that decision-makers are predominantly found on the opposite side of this dense congregation with limited direct ties with the nutrition professionals. In the middle of these two groups and with the highest *betweenness centrality* (see Table 1) of all actors in the network is a General Health Professional from a non-government organisation (NGO). This person is in a key brokerage role (Broker 1). The authors acknowledge that the reported *betweenness centrality* appears high at face value, but the size of the network, coupled with relatively low density and modest average path distance make the resulting betweenness less surprising. To make these reported figures more easily comparable to other network studies, a column normalising (scaled between zero and one) the reported betweenness centrality scores is also provided in Table 1, which further illustrates the prominence of the two Brokers that are focused on in this paper.

Other actors with high *betweenness centrality* can be seen in Table 3. A Nutrition Professional Academic has the second highest ranking (Broker 2); however, when examining Fig. 1 you can see this individual is surrounded by the cluster of nutrition professionals. This indicates Broker 2 is taking on a brokering role amongst nutrition professionals rather than brokering relations with decision-makers. The three remaining actors in the top 5 brokers have much lower *betweenness centrality* scores which correlate to their lower *in-degree* scores.

## Discussion

Relationships between policymakers and interest groups can often occur behind closed doors and are not easily visible. In this paper we have analysed data from a range of nominated influential actors that make up the nutrition policy network in Australia. There is a specific focus on brokers as their structural position is assumed to give them a decisive impact on policy outcomes.

The network reveals two key brokers with the General Health NGO broker (referred to as Broker 1) in a much more prominent (strategic) position for influencing policymakers, than the Nutrition Professional Academic broker (Broker 2). The network data demonstrated that Broker 1 held a commanding position with respect to network connections and strategic position within the network that no other actor was able to match. This position gives this individual more power by allowing them to better control the flow of important resources such as information to and from other members of the network and brokering new relationships which in turn can set and shape agendas. A potential downside of actors within centrally located network positions is that while they can leverage their centrality to link actors with resources and other constructive relationships within their network, they also have the capacity to prevent other actors from participating in certain policymaking decisions or agenda setting decisions [27].

Broker 2 (Nutrition Professional Academic) has a clear brokering role among nutrition professionals. This is an important role for ensuring the flow of information between nutrition professionals in government, academia and within NGO’s. However, the overall network position of Broker 2 is not as close to policy decision-makers, meaning that it requires greater investment of time and relational capital for Broker 2 to access and link information to decision-makers when compared to Broker 1. So, while Broker 2 is highly prominent within the policymaking network, Broker 2’s strategic position is not potentially as effective as that of Broker 1.

Other Brokers listed in Table 2 scored far lower in *betweenness centrality* than the top two Brokers. This dramatically lower *betweenness centrality* is more a product of their position in the network (with respect to the distance from central decision makers) and their linking to fewer discrete sub-groups in the network (and subsequently lower *degree* scores). This translates to a far lower visibility to others within the nutrition network for these lower ranked Brokers. This reduced visibility is further triangulated in the data from the imbalance between the lower ranked Broker’s *in-degree* and *out-degree*. That is, a high *out-degree* demonstrates the Broker identifies themselves as having active relationships with many others in the network (typically across many categories of actors), whereas the lower *in-degree* indicates that few in the network see the Broker as playing an influential role in the policy making network (i.e. only seen as influential to particular categories within the network). This highlights the value in the social network analysis methodology in making the invisible networks visible. For example, Broker 4, from the food industry, was well connected with decision-makers yet only one nutrition professional nominated this person. While Broker 4 has fewer total connections, they are extremely well-placed to access and potentially influence decision makers. Also, a key government decision maker (Broker 5) had very few people in the network nominate them as an influential direct connection, indicating a lack of awareness of the importance of this individual in decision-making or an inability to develop a relationship with a bureaucrat this senior. This presents an opportunity for actors to review their network connections and their position within the policymaking networks to ensure they remain effective within the network.

At face value, the network pictured in Fig. 1 appears quite dense, which makes the prominence of Broker 1 and Broker 2, as illustrated by their exceptionally high *betweenness centrality* reported in Table 2, appear out of place. That is, a relatively dense network would typically have many pathways available for actors to access other actors in the network. This increased number of pathways would normally result in downward pressure on the *betweenness centrality* for actors in the network. However, the relatively low network density (0.009539) indicates that the network is at least somewhat fragmented, with a previous analysis of the data [16] confirming this by identifying several distinct clusters within the overall nutrition policy network. Relationships between the clusters were often mediated by Brokers 1 and 2, helping to keep the average geodesic path distance (number of steps to get from one actor to another) down to a modest 3.29007 (maximum of 7). Consequently, Brokers 1 and 2 make it far easier for actors in one area of the network to access actors in another, hence their extraordinarily high reported *betweenness centrality* scores.

The two top-ranked Brokers have extremely high *degree centrality* (both *in* and *out degree*) compared with the remaining participants. This demonstrates how well-known these two Brokers are within the nutrition policy network, as well as how effective they are at linking to others within the network, compared to the remaining policy actors. This is a common phenomenon in SNA known as preferential attachment [32]. Popular actors often tend to become more popular because they have high visibility to begin with. This results in degree distributions which are often positively skewed to a small number of actors with very high *degree* and many actors with lower *degrees* [32].

Of interest, is that, despite Broker 1’s advantageous brokerage position, the limited number of policy outcomes for nutrition suggests that the actor, and potentially the network as a whole, has a limited impact on nutrition policy action at a federal level on Australia. This may reflect the complexity of nutrition as an issue or it may reflect the lack of focus on nutrition by Broker 1 due to competing priorities associated with their general health role. It may also point to the fact that brokerage in isolation may not be as influential in nutrition policy as other network measures such as direct access to decision makers as discussed in a previous paper by the authors. By extension, this indicates that a strategic brokerage position within a decision making network is not enough to influence policy, and that many other factors are likely at play including ideology and beliefs of decision-makers, the salience of the issue, opposing pressure from the food industry, unsupportive institutional norms and a lack of public will [17, 37, 38]. Furthermore despite the close ties amongst the nutrition community in Australia, previous studies have shown that this does not necessarily translate into expert consensus around nutrition issues [17]. This lack of consensus hinders the development of strong, advocacy groups and therefore decreases the likelihood of policy change [14].

Importantly these results show that brokers may perform different roles in the network to what some scholars have traditionally assumed, which is that policy brokers are synonymous with policy entrepreneurs [33]. Policy entrepreneurs act in an opportunistic and strategic way to promote their interests so the final outcome reflects their policy preferences [39]. However, the current role of the Broker 1 in nutrition policy may be more in line with the policy broker definition provided by the Advocacy Coalition Framework [14] where brokers are seen as actors that seek stability through connecting interest groups that differ in their beliefs. In these situations, policy brokers can intervene by promoting conciliatory policy solutions and by mediating trust [40].

Another important role that brokers hold within policy networks is to steward policy discussions of the more discrete actors that fall within their personal network. Consequently, there is an opportunity for brokers in nutrition policy to act not only as aggregators and conduits of information, but as mentors to their sub-networks as well. It may be worth considering alternate ways to support key brokers in the nutrition network or utilise their strategic position to improve the effectiveness of nutrition advocacy and/or improve the ability of more discrete actors to align their efforts in a more coordinated way. This could take the form of a broker mentoring a group of actors to help them develop key relationships and share the burden of linking discrete actors with decision-makers. Alternatively a good investment would be creating more opportunities for establishing linkages between actors within the network to provide better visibility of the range of initiatives taken by actors who would identify as discrete.

## Conclusion

This study has added value in terms of understanding influential actors and power in an environment where policy is frequently made by a diverse range of actors whose influence is often derived from access to political and social capital. The results of this study suggest there are two dominant brokers of the nutrition policy network in Australia. However their position in the network means their brokerage roles have different purposes and different levels of influence for policymaking. In the highly contested nutrition policy space, many other factors are likely at play including ideology and beliefs of decision-makers, unappealing solutions, lack of consensus from the nutrition community, opposing pressure from the food industry and lack of public will. In addition, this study has also highlighted that many nutrition advocates are not aware of and do not have direct links with other key influential individuals. As networks are not static, there are opportunities for advocates to change the current network by ensuring advocates for nutrition policy change move into more advantageous positions. There is a role for the key brokers to utilise their position to improve the effectiveness of nutrition advocacy and/or improve the ability of more discrete actors to align their efforts in a more coordinated way.

### Limitation

This study has certain limitations. Firstly, brokerage is only one aspect of actor influence in a policy network. Aspects beyond relationships can impact on policy outcomes including public will, beliefs and values of decision-makers, party policy, and media coverage of an issue. Incorporating qualitative data from key network actors could provide further insight into power and influence in nutrition policy. Secondly, this is a cross-sectional design and therefore limits the ability to infer causality or temporality, and it may also mask potential shifts in power over time as well as underlying power. If additional resources were available a longitudinal analysis would improve the study. Finally, while the response rate for this study was high for an elite network of this size, a higher response rate, particularly from the political sector, would have provided greater confidence in our data.

## Additional file

Additional file 1: Survey used to gather data regarding interest groups and individuals and their influence on nutrition policy in Australia. (DOCX 31 kb)

## Abbreviations

NGO: Non-government organisation
