# Supplementary material for: Joining the dots: the role of brokers in nutrition policy in Australia
Source: BMC Public Health. 2017 Apr 8;17:307. doi: 10.1186/s12889-017-4217-8 (PMC5385063; doi:10.1186/s12889-017-4217-8)
Supplement: Additional file 1: — Survey used to gather data regarding interest groups and individuals and their influence on nutrition policy in Australia. (DOCX 31 kb) [file 12889_2017_4217_MOESM1_ESM.docx]

| [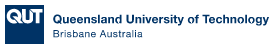](http://www.qut.edu.au/) |  |
| --- | --- |
| **An exploration of interest groups and their influence on nutrition policy in Australia**  **QUT Ethics Approval Number 1400000857** | |

I am conducting a survey to identify the most influential people in **national** nutrition policy in Australia. Please nominate the people you feel are influential in national nutrition policy in Australia, using the following definition:

The person demonstrates a capacity to do one or more of the following:

- shape ideas about policy
- initiate policy proposals
- substantially change or vet other’s proposals, or
- substantially affect implementation of policy related to food and nutrition

Please nominate **as many or as few people** that you can think of. If you are unable to name a person, please name the position or organisation you feel meets the above criteria. Please note you do not need to provide contact details for the person or organisation.

|  | Name of person | Position | Organisation | Are you in **direct** contact with this person? Y/N | How often are you in **direct** contact? Please tick or leave blank | | | | | |
| --- | --- | --- | --- | --- | --- | --- | --- | --- | --- | --- |
|  |  |  |  |  | Daily | Weekly | Monthly | Quarterly | 6 monthly | Yearly |
| A1 |  |  |  |  |  |  |  |  |  |  |
| A2 |  |  |  |  |  |  |  |  |  |  |
| A3 |  |  |  |  |  |  |  |  |  |  |
| A4 |  |  |  |  |  |  |  |  |  |  |
| A5 |  |  |  |  |  |  |  |  |  |  |
| A6 |  |  |  |  |  |  |  |  |  |  |
| A7 |  |  |  |  |  |  |  |  |  |  |
| A8 |  |  |  |  |  |  |  |  |  |  |
| A9 |  |  |  |  |  |  |  |  |  |  |
| A10 |  |  |  |  |  |  |  |  |  |  |
| A11 |  |  |  |  |  |  |  |  |  |  |
| A12 |  |  |  |  |  |  |  |  |  |  |
|  | Name of person | Position | Organisation | Are you in **direct** contact with this person? Y/N | How often are you in **direct** contact? Please tick | | | | | |
|  |  |  |  |  | Daily | Weekly | Monthly | Quarterly | 6 monthly | Yearly |
| A13 |  |  |  |  |  |  |  |  |  |  |
| A14 |  |  |  |  |  |  |  |  |  |  |
| A15 |  |  |  |  |  |  |  |  |  |  |
| A16 |  |  |  |  |  |  |  |  |  |  |
| A17 |  |  |  |  |  |  |  |  |  |  |
| A18 |  |  |  |  |  |  |  |  |  |  |
| A19 |  |  |  |  |  |  |  |  |  |  |
| A20 |  |  |  |  |  |  |  |  |  |  |

If you think about national nutrition policy specifically around **Indigenous nutrition**, who would you list as influential? If repeating names from the original list, please write the number associated with that name, for example, A3.

|  | Name of person | Position | Organisation | Are you in **direct** contact with this person? Y/N | How often are you in **direct** contact? Please tick | | | | | |
| --- | --- | --- | --- | --- | --- | --- | --- | --- | --- | --- |
|  |  |  |  |  | Daily | Weekly | Monthly | Quarterly | 6 monthly | Yearly |
| B1 |  |  |  |  |  |  |  |  |  |  |
| B2 |  |  |  |  |  |  |  |  |  |  |
| B3 |  |  |  |  |  |  |  |  |  |  |
| B4 |  |  |  |  |  |  |  |  |  |  |
| B5 |  |  |  |  |  |  |  |  |  |  |
| B6 |  |  |  |  |  |  |  |  |  |  |
| B7 |  |  |  |  |  |  |  |  |  |  |
| B8 |  |  |  |  |  |  |  |  |  |  |
| B9 |  |  |  |  |  |  |  |  |  |  |
| B10 |  |  |  |  |  |  |  |  |  |  |
| B11 |  |  |  |  |  |  |  |  |  |  |

If you think about national nutrition policy around **school gardening and cooking programs**, who would you list as influential? If repeating names from a previous list, please write the number associated with that name, for example, B3.

|  | Name of person | Position | Organisation | Are you in **direct** contact with this person? Y/N | How often are you in **direct** contact? Please tick | | | | | |
| --- | --- | --- | --- | --- | --- | --- | --- | --- | --- | --- |
|  |  |  |  |  | Daily | Weekly | Monthly | Quarterly | 6 monthly | Yearly |
| C1 |  |  |  |  |  |  |  |  |  |  |
| C2 |  |  |  |  |  |  |  |  |  |  |
| C3 |  |  |  |  |  |  |  |  |  |  |
| C4 |  |  |  |  |  |  |  |  |  |  |
| C5 |  |  |  |  |  |  |  |  |  |  |
| C6 |  |  |  |  |  |  |  |  |  |  |
| C7 |  |  |  |  |  |  |  |  |  |  |
| C8 |  |  |  |  |  |  |  |  |  |  |
| C9 |  |  |  |  |  |  |  |  |  |  |
| C10 |  |  |  |  |  |  |  |  |  |  |
| C11 |  |  |  |  |  |  |  |  |  |  |

If you think about national nutrition policy around **front of pack food labelling**, who would you list as influential? If repeating names from a previous list, please write the number associated with that name, for example, B3.

|  | Name of person | Position | Organisation | Are you in **direct** contact with this person? Y/N | How often are you in **direct** contact? Please tick | | | | | |
| --- | --- | --- | --- | --- | --- | --- | --- | --- | --- | --- |
|  |  |  |  |  | Daily | Weekly | Monthly | Quarterly | 6 monthly | Yearly |
| D1 |  |  |  |  |  |  |  |  |  |  |
| D2 |  |  |  |  |  |  |  |  |  |  |
| D3 |  |  |  |  |  |  |  |  |  |  |
| D4 |  |  |  |  |  |  |  |  |  |  |
| D5 |  |  |  |  |  |  |  |  |  |  |
| D6 |  |  |  |  |  |  |  |  |  |  |
| D7 |  |  |  |  |  |  |  |  |  |  |
| D8 |  |  |  |  |  |  |  |  |  |  |
| D9 |  |  |  |  |  |  |  |  |  |  |
| D10 |  |  |  |  |  |  |  |  |  |  |
| D11 |  |  |  |  |  |  |  |  |  |  |

**Thank you for your time.**
